# HIV-1 Receptor Binding Site-Directed Antibodies Using a VH1-2 Gene Segment Orthologue Are Activated by Env Trimer Immunization

**DOI:** 10.1371/journal.ppat.1004337

**Published:** 2014-08-28

**Authors:** Marjon Navis, Karen Tran, Shridhar Bale, Ganesh E. Phad, Javier Guenaga, Richard Wilson, Martina Soldemo, Krisha McKee, Christopher Sundling, John Mascola, Yuxing Li, Richard T. Wyatt, Gunilla B. Karlsson Hedestam

**Affiliations:** 1 Department of Microbiology, Tumor and Cell Biology, Karolinska Institutet, Stockholm, Sweden; 2 IAVI Neutralizing Antibody Center at The Scripps Research Institute, La Jolla, California, United States of America; 3 Department of Immunology and Microbial Science, The Scripps Research Institute, La Jolla, California, United States of America; 4 Vaccine Research Center, National Institute of Allergy and Infectious Diseases, National Institutes of Health, Bethesda, Maryland, United States of America; Miller School of Medicine, United States of America

## Abstract

Broadly neutralizing antibodies (bNAbs) isolated from chronically HIV-1 infected individuals reveal important information regarding how antibodies target conserved determinants of the envelope glycoprotein (Env) spike such as the primary receptor CD4 binding site (CD4bs). Many CD4bs-directed bNAbs use the same heavy (H) chain variable (V) gene segment, VH1-2*02, suggesting that activation of B cells expressing this allele is linked to the generation of this type of Ab. Here, we identify the rhesus macaque VH1.23 gene segment to be the closest macaque orthologue to the human VH1-2 gene segment, with 92% homology to VH1-2*02. Of the three amino acids in the VH1-2*02 gene segment that define a motif for VRC01-like antibodies (W50, N58, flanking the HCDR2 region, and R71), the two identified macaque VH1.23 alleles described here encode two. We demonstrate that immunization with soluble Env trimers induced CD4bs-specific VH1.23-using Abs with restricted neutralization breadth. Through alanine scanning and structural studies of one such monoclonal Ab (MAb), GE356, we demonstrate that all three HCDRs are involved in neutralization. This contrasts to the highly potent CD4bs-directed VRC01 class of bNAb, which bind Env predominantly through the HCDR2. Also unlike VRC01, GE356 was minimally modified by somatic hypermutation, its light (L) chain CDRs were of average lengths and it displayed a binding footprint proximal to the trimer axis. These results illustrate that the Env trimer immunogen used here activates B cells encoding a VH1-2 gene segment orthologue, but that the resulting Abs interact distinctly differently with the HIV-1 Env spike compared to VRC01.

## Author Summary

The development of an HIV-1 vaccine that stimulates the production of antibodies capable of neutralizing diverse circulating HIV-1 strains remains a global priority. Studies have shown that broadly neutralizing antibodies (bNAbs) isolated from HIV-1 infected individuals can protect against infection in non-human primates and, in some cases, reduce viremia after already established infection. An intriguing feature of one class of bNAbs directed against the primary receptor binding site of HIV-1, the CD4 binding site (CD4bs), is that they are encoded by the same heavy chain gene segment, which encodes critical contacts for this class of Ab. Here, we asked if HIV-1 Env vaccination activates B cells that encode the rhesus macaque orthologue of this gene segment and if so what the genetic and structural properties of such antibodies are. We isolated a set of monoclonal antibodies encoded by this gene segment and demonstrate that one such Ab, GE356, binds the receptor binding site in a manner that is distinctly different from the mode of interaction of CD4bs-directed bNAbs. These results provide a possible explanation for the lack of broadly neutralizing activity following vaccination, even when antibodies encoded by gene segments frequently used in bNAbs are elicited.

## Introduction

The neutralization resistant properties of primary HIV-1 isolates, conferred by structural features of the HIV-1 Env spike, present a major hurdle for developing a protective vaccine [1]–[4]. Despite the highly evolved capacity of the functional HIV-1 Env spike to evade recognition by Abs generated at high levels in most infected individuals, potent and broad serum-neutralizing activity develops in some HIV-1 infected individuals after years of active viral replication [5]. Once such responses develop, a single or a few Ab specificities can be responsible for the neutralizing activity present in the polyclonal serum [6]–[8]. Studies aimed at defining Ab specificities that mediate broad and potent neutralizing activity in HIV-1 infected individuals have led to the isolation of multiple broadly neutralizing antibodies (bNAbs) [9]–[13]. While bNAbs do not ameliorate HIV-1 replication within the individual whom they arise, they can protect against de novo infection as shown in experimental passive transfer-challenge studies in non-human primates (NHPs) [14]–[20]. Recently, the capacity of highly potent bNAbs to suppress already established viremia in NHPs was reported [21], [22] illustrating the potential of bNAbs for both clinical prophylaxis and therapy [23], [24].

The demonstration that several of the most effective bNAbs are directed against the CD4bs, a functionally conserved region on the exterior envelope glycoprotein, gp120, has elevated the attractiveness of this target as a neutralizing Ab determinant [9], [12], [25], [26]. A feature of the broadly neutralizing CD4bs-directed monoclonal antibodies (MAbs) is their extreme level of somatic hypermutation (SHM), which can be as much as 30–36% divergent from the respective putative germline nucleotide sequence [9], [12], [13]. Furthermore, many of the broadly neutralizing CD4bs-directed Abs described so far display restricted variable heavy chain (VH) usage with many, but not all, utilizing the VH1-2*02 gene segment [9], [12], [13], [27]. Fab structures derived from several CD4bs-directed, VH1-2*02-using MAbs, including VRC01, VRC-PG04 and 3BNC117, were solved in complex with the gp120 core, revealing a mode of binding that closely mimics that of CD4 [9], [13], [26]–[28]. Besides extensive SHM and restricted VH usage, several of the broadly neutralizing VH1-2*02-using CD4bs-directed Abs use light chains with a 5-amino acid third complementarity determining region (CDR3), which, based on structural analyses, is predicted to be critical to avoid clashes with the Ab cognate epitope on gp120. These bNAbs also often possess a deletion in their LCDR1, once again to avoid clashes with elements of gp120 [26], [27], [29].

In contrast to the humoral immune response elicited during chronic HIV-1 infection, which can persist for 10 years or longer, the more transient HIV-1 vaccine-induced Ab responses generate lower levels of SHM [30]. Also in contrast to chronic infection, broadly neutralizing MAbs have so far not been isolated following Env vaccination, with one exception of a CD4bs-directed Ab isolated following Env vaccination of a llama [31]. However, llamas differ from primates in that they naturally generate free, low molecular weight VH antibodies capable of accessing privileged sites not accessible to larger bivalent immunoglobulin molecules generated in primates. The lower mass of this llama Ab likely contributes to its neutralization breadth and potency [31].

To begin to understand the limitations of vaccine-induced Ab responses against HIV-1 Env, we recently established approaches for genetic and functional analyses of Ab responses elicited in NHPs [30]. We demonstrated that CD4bs-directed Abs capable of neutralizing selected HIV-1 isolates are elicited in rhesus macaques immunized with soluble gp140-F trimers [32], offering a pathway forward for more detailed analyses of such B cell and antibody responses. Furthermore, we established a database of the concurrent rhesus macaque VH and variable light chain (VL) germline sequences and determined sequence homologies between macaque and human VH germline sequences [30], which greatly facilitates future genetic analyses of antibody repertoires in NHPs.

Here, we identified the VH1.23 gene segment to be the macaque germline VH sequence most closely related to the human VH1-2 segment, with 92% homology to the human VH1-2*02 allele. We isolated VH1.23-using CD4bs-directed MAbs elicited by subunit Env trimer vaccination and describe the genetic and functional properties of a panel of such MAbs. For the most potent of these MAbs, GE356, an Ab minimally modified by somatic hypermutation (SHM) as shown by analysis of its putative germline sequence, a Fab structure was obtained. Structural analysis allowed the interaction between the GE356 Fab and gp120 to be investigated through epitope and paratope alanine scanning, as well as modeling analysis of GE356 binding to monomeric or trimeric HIV-1 Env structures. These data showed that the VH1.23-using CD4bs-directed GE356 MAb interacted in a similar manner with its target epitopes as observed for two previously described vaccine-induced macaque VH4-using CD4bs-directed Abs [33]. These data suggest that the cognate CD4bs presented by these Env trimers is highly immunogenic, but stimulates CD4bs-directed Abs of diverse VH usage that are suboptimal in orientation and display detectable but limited breadth of neutralization.

## Materials and Methods

### Ethics statement

The three-year-old rhesus macaque (Macaca mulatta) of Chinese origin, designated F128, sampled for this study was described elsewhere [32]. Animals were housed at the animal facility of the Astrid Fagraeus Laboratory at the Swedish Institute for Infectious Disease control. Housing and care procedures were in compliance with the provisions and general guidelines of the Swedish Board of Agriculture, which are even stricter than the adopted European Directive 2010/63/EU on the protection of animals used for the scientific purposes, and the facility has been assigned an Animal Welfare Assurance number by the Office of Laboratory Animal Welfare (OLAW) at the National Institute of Health. All procedures were approved by the Local Ethical Committee on Animal Experiments (Stockholms Norra Djurförsöksetiska Nämnd) (ethical permit number N85/09 and N32/12). The animals were housed in pairs in 4-m^3^ cages and all primate rooms have daylight. The rooms are enriched to give them possibility to express their physiological and behavioral needs. All primates have bedding (wood chips, straw, hay, shredded paper, etc) on at least a portion of the floor in which foraging for small edibles (seeds, rice etc) is an option. Furthermore, a number of enrichment devices and puzzles are filled or added/removed in addition to destructible toys (wood sticks; tennis balls; cleaned shampoo bottles etc). They are offered variable items to stimulate several senses (smell; touch; sight; hearing). Certain food items are chopped in order to deliver pieces small enough to be able to rinse them out of the cages without opening them. In other instances, fruit and vegetables are served whole. They get a bathtub at least once a week and have access to mirrors in which they can monitor the environment without having to resort to direct eye contact.

They were habituated to the housing conditions for >6 wk before the start of the experiment and subjected to positive reinforcement training to reduce the stress associated with experimental procedures. All immunizations and blood samplings were performed under sedation with 10 mg/kg ketamine i.m. (100 mg/ml Ketaminol; Intervet). The macaques were weighed at each immunization or sampling occasion.

### Sampling

A detailed description of the immunization experiment was described previously [32], which essentially consists of gp140-F trimers in adjuvant (Abisco-100 with CpG-C) inoculated five times at monthly intervals. The MAbs described in the current paper were isolated from a bleed collected 1 week after the second vaccine inoculation.

### Protein expression and purification

The soluble gp140-F trimers [34] used as the immunogen were produced by transient transfection of a CMV-driven expression plasmid into FreeStyle 293F suspension cells (Invitrogen) as previously described [35]. In brief, the gp140-F trimers are soluble cleavage-defective oligomers derived from the primary HIV-1 R5 isolate, YU2, possessing a stabilizing C-terminal heterologous trimerization motif derived from T4 bacteriophage fibritin, known as foldon (F). The gp140-F trimers were purified by sequential lectin and chelation affinity chromatography. Env ligands used in binding studies, gp120 and gp120 core (core, previously referred to as V3S) were purified by a 17b MAb-coupled protein A-Sepharose column [36]. TriMut core and TriMut-368/70 were purified by lentil-lectin and gel filtration chromatography. The biotinylated gp140-F and gp140-F-D368R probes used for Env-specific cell sorting and enrichment of CD4bs-directed memory B cells flow cytometry were purified by lectin chromatography and IMAC. Both probes carried an Avi-tag signal for site-specific biotinylation to the C-terminus of gp140-F [37] and biotinylation was performed with biotin ligase Bir A (Avidity, Denver, CO). All gp120 and gp140 proteins, with exception to SF162 gp120 (IAVI-NAC Repository), were on the YU2 background, while the core proteins were based on HXBc2.

For crystallization studies, plasmids encoding the heavy and light chains for the GE356 Fab were transfected and the protein was expressed in Freestyle 293F cells. The plasmid for the heavy chain encoded for a 6×-His tag at the C-terminus of the polypeptide. The protein was expressed over a period of 4–5 days, 37°C with shaking at 225 rpm. The media containing the protein was separated from the cells by centrifugation at 4000 rpm for 20 min. The supernatant was applied to a Ni column pre-equilibrated with wash buffer containing 10 mM Tris, pH 7.5, 150 mM NaCl and 10 mM imidazole. The column was subsequently washed with 10 column volumes of wash buffer, followed by another wash, with wash buffer containing 30 mM imidazole. The Fab protein was eluted in 5 column volumes of wash buffer containing 300 mM imidazole. The protein was further purified using Superdex 200 size exclusion column (GE Healthcare) in 10 mM Tris, pH 7.5, 150 mM NaCl.

### Single cell sorting by flow cytometry

CD4bs-specific memory B cells were sorted at single cell density with a three-laser FACSAria cell sorter (BD Biosciences), as described previously. Cryopreserved monkey PBMCs were thawed and stained with fluorescently labeled Abs for cell surface markers as well as Env probes as previously described [30]. The fluorescently labeled antibodies for cell surface markers include CD3, CD8, CD14, CD20, IgG, CD27, and IgM. Aqua Blue (Invitrogen) was also included to exclude dead cells. To selectively sort CD4bs-specific memory B cells, we used the TriMut and TriMut368/370 probes biotinylated at the C-termini. The biotin-labeled TriMut probe was conjugated to streptavidin-allophycocyanin (SA-APC) (Invitrogen), and TriMut368/370 to extravidin-phycoerythrin (EA-PE) (Sigma) to yield TriMut-APC and TriMut368/370-PE [12]. Each Env probe used in the staining cocktail was at a final concentration of 4 µg/ml. CD4bs-specific memory B cells were defined as CD3−, CD8−, Aqua Blue−, CD14−, CD20+, IgG+, CD27+, IgM−, TriMut+, and TriMut368/370-, and were collected into single wells of 96-well PCR plates containing 20 µl of cell lysis buffer [12] and stored at −80°C prior to RT-PCR.

### RT-PCR of IgG genes, cloning and expression

The IgG heavy chain gene transcripts of single sorted NHP memory B cells were amplified by RT-PCR and cloned into eukaryotic expression vectors to produce full IgG1 Abs, with Ig-specific PCR conditions, primers, and expression vectors as described [38]. The corresponding light chain gene transcripts of the single sorted NHP memory B cells were amplified with a protocol optimized for rhesus macaque V(D)J sequences [39]. As shown in these prior studies, these primer sets allow amplification of a diverse set of V gene segments. The primers that were used for cloning the PCR product into the expression vector were 5′AgeI VH1/5 (CTGCAACCGGTGTACATTCCGAGGTGCAGCTGGTGCAG) and 3′SalI JH1/2/4/5 (TGCGAAGTCGACGCTGAGGAGACGGTGACCAG) with 5′AgeI VH1/5 introducing the Q to E amino acid change at position one of the heavy chain variable region. For expression, equal amounts of heavy- and light-chain plasmid DNAs (15 µg of each) were transfected with 30 µl of FreeStyle Max reagent (Invitrogen) into 30 ml of FreeStyle 293F cells at a density of 1 million cells/ml. The cell culture supernatant containing the secreted IgG was harvested 7 days after transfection and purified by protein A–Sepharose columns (GE Healthcare). The MAbs characterized in the current study have been submitted to the GenBank database under accession numbers KF939869-KF939878 (KF939869: GE331 HC, KF939870: GE356 HC, KF939871: GE453 HC, KF939872: GE443 HC, KF939873: GE460 HC, KF939874: GE443 KC, KF939875: GE331 KC, KF939876: GE453 KC, KF939877: GE356 LC, KF939878: GE460 LC).

### Rhesus Ab heavy chain variable region gene analysis

**Figure 1:**
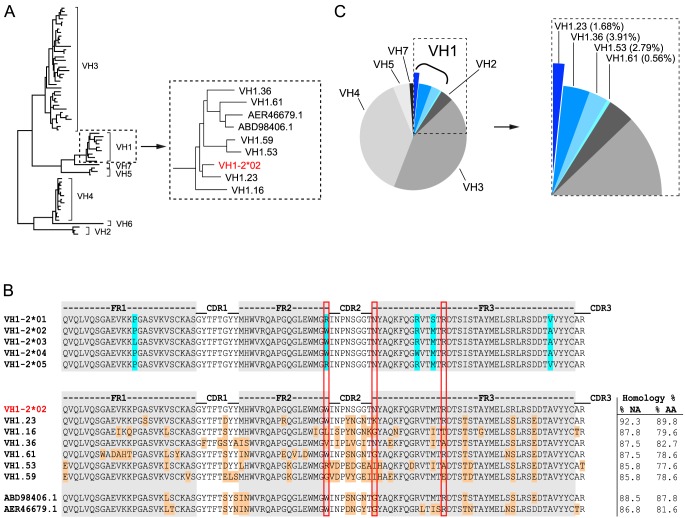
NHP VH1 gene segment usage. (A) Phylogenetic representation of known rhesus macaque VH germline sequences. The VH1 family is boxed and shown in a magnified view to highlight the VH1 rhesus macaque germline sequences in relation to the human VH1-2*02 germline sequence (red). (B) Sequence alignments of all known human VH1-2 alleles are shown in the top panel. Framework (FR) regions are shaded in gray with the amino acid differences between the alleles highlighted in blue. Sequence alignments of the human VH1-2*02 germline sequence with the known rhesus VH1 germline sequences are shown in the lower panel. Differences between human VH1-2*02 and the rhesus germline sequences are highlighted in orange and the percentage nucleic acid (NA) and amino acid (AA) homologies are indicated to the right after each germline sequence. The W50/N58/R71 motif, which is typically present in VH1-2*02-using bNAbs, is highlighted by the vertical red boxes. (C) Pie-chart indicating the family usage of 179 VH sequences isolated from animal F128. Sequence “slices” belonging to the VH1 family are boxed and highlighted in different shades of blue. The percentage of sequences annotated to a given germline gene segment within the VH1 family is indicated in parentheses in the magnified view to the right.

For phylogenetic analysis, the germline VH1-2*02 gene from humans (IMGT) and all known rhesus macaques germlines as described previously [30] were aligned with MUSCLE (version 3.7) iterated 50 times. Alignment gaps were removed with Gblocks. Phylogeny was calculated with PhyML (version 3.0), and maximal likelihood trees were rendered using the HKY85 substitution model and bootstrapping (200 to 500 replicates) with TreeDyn (version 198.3) [40]. The trees were then graphically edited with Dendroscope [41]. SHM calculations were performed on the VH region between codons 1 and ∼99 (IMGT count; conserved cysteine, marking the end of the V region) through alignment to the previously published rhesus genome [42]. CDR3 lengths were determined by means of IMGT/V-QUEST. SHM calculations were performed on the VH regions by alignment to the appropriate germline sequences. The VH1.23 germline sequence in animal F128 was determined by genomic sequencing. In brief, DNA was isolated from pre-vaccination frozen PBMCs (Qiagen QIAamp DNA mini kit) and sequences were amplified using two independent primer sets (Fig. S2A). Primer set 1 (PCR1) consisted of MMUL VH1.23 fw (ggtggcctgagctatgaaatacctg) in combination with MMUL VH1.23 rev (acctggcataattcccagtgaccc) aligning to sequences present in the non-coding genomic DNA flanking the VH1.23 reading frame. Primer set 2 (PCR2) consisted of 5′VH1A.se (tggcagcagctacaggtaa) in combination with VH1.23 AS-A (gcttcttttcgtcaattacttaacc), where the forward primer aligned to a sequence in leader 1 of VH1.23 and the reverse primer to a sequence in the non-coding DNA. These primers were chosen to ensure amplification of VH1.23 sequences arising from non-rearranged germline DNA and not recombined V(D)J sequences. The PCR fragments were ligated into a cloning vector (CloneJET, Thermo Scientific) and bacteria were transformed (XL10-Gold Ultracompetent cells, Agilent Technologies) for analysis of multiple colonies from independent PCR products (Fig. 1B).

### Virus neutralization assays

Neutralization assays were performed with an HIV-1 Env pseudovirus assay and TZM-bl target cells [43]. To determine the Ab concentration that resulted in a 50% reduction in relative luciferace units, we performed serial dilutions of the Abs and fitted the neutralization dose response curves by nonlinear regression with a four-parameter Hill slope equation programmed into JMP statistical software (JMP 5.1, SAS Institute Inc.) or into GraphPad Prism 6 (GraphPad Software Inc.). The results are reported as Ab concentration resulting in 50% virus neutralization (IC_50_). Diverse HIV-1 virus isolates, including viruses from clades A, B, and C, were used in the neutralization assays. The sources of the Env-encoding plasmids were described previously [32].

### Characterization of Env binding

The MAbs were tested for binding using Reacti-Bind (Pierce) ELISA plates coated at 2 µg/ml with gp140-F, gp140-F-D368R, TriMut, or TriMut-368/70 in PBS at 4°C overnight. After blocking in blocking buffer (PBS containing 2% non-fat milk) or B3T buffer (150 mM NaCl, 50 mM tris-HCl, 1 mM EDTA, 3.3% FBS, 2% bovine albumin, and 0.07% Tween 20), the MAbs were added and incubated for 1 hour at 37°C. Binding was detected by secondary horseradish peroxidase (HRP)–conjugated anti-human Fcγ (Jackson ImmunoResearch) at 1∶10,000 for 1 hour. The signal was developed by addition of 3,3′,5,5′-tetramethylbenzidine (TMB) substrate (SureBlue; KPL) for 10 min. Reactions were terminated with 1 N sulfuric acid, and the optical density (OD) was read at 450 nm. Between each incubation step, the plates were washed six times with PBS containing 0.05% Tween 20.

### Ab binding kinetic analysis

The kinetics of NHP and human MAb binding to a panel of Env variants were assessed with an Octet RED96 system (ForteBio) with Bio-Layer Interferometry (BLI) in a 96-well format following the manufacturer's instructions as described previously [30]. Abs at 10 µg/ml in binding buffer (PBS/0.2% Tween 20) were captured on the surface of the anti-human IgG Fc capture biosensors (ForteBio) for 1 minute, followed by 1 minute wash in binding buffer to establish a new baseline signal, and the biosensor is then immersed in wells containing the Env variants which are 2-fold serially diluted in binding buffer at an initial starting concentration of 250 nM. Ab-Env association rate (on-rate) and the dissociation rate (off-rate) were measured over a 2-min interval, respectively. K_D_ values (in nanomolar) were calculated as off-rate/on-rate.

### Epitope mapping

A panel of 27 gp120 Ala mutants containing alterations known to affect CD4 binding or recognition by a set of CD4bs-directed MAbs was selected. The Ala mutations were generated previously in the context of the full-length JRCSF gp160 expression plasmid [44]. The Env plasmids were individually co-transfected into 293T cells together with a plasmid containing the remaining HIV structural genes to produce Env pseudoviruses as described previously [43]. For binding analysis, the gp120 was released from the pseudovirus by detergent lysis and then captured by the sheep anti-gp120 C5 Ab, D7324 (Aalto Bio Reagents) previously coated into ELISA wells of the 96-well plate. After washing, binding to gp120 was assessed for the human and NHP MAbs with an anti-Fc HRP secondary Ab and the TMB substrate. The level of binding by the human MAb 2G12 was used to normalize the variant gp120 expression levels. The effect of a given Ala mutation on Ab binding was represented by apparent affinity (avidity) relative to the binding level to wild-type (WT) gp120, calculated with the formula [(EC_50__WT/EC_50__mutant)/(EC_50__WT for 2G12/EC_50__mutant for 2G12)]×100, where EC_50_ is the median effective concentration, as previously described [45].

### Paratope mapping

A panel of 53 GE356 and 41 VRC01 Ala mutants containing alterations at every amino acid position in the CDR heavy and light chain regions were purchased from Genscript (using IMTG DomainGapAlign and Wu et al. [12]). Amino acid numbering was performed according to the Kabat system. The Ala mutations were generated in the context of the full-length heavy or light chains and for expression, equal amounts of heavy- and light-chain plasmid DNAs (15 µg of each) were transfected with 0.1 ml of 293Fectin into 30 ml of FreeStyle 293F cells at a density of 1 million cells/ml. The cell culture supernatant containing the secreted IgG was harvested 7 days after transfection and purified by protein A Sepharose columns (GE Healthcare). The antibodies containing the single Ala mutations were tested for neutralization activity and gp120 binding as described above.

### Crystallization and MAb structure determination

The GE356 Fab was incubated in a sitting drop format in 1∶1 ratio with sparse matrix crystallization solutions. The protein crystallized in 0.2 M Tri-lithium citrate, 20% PEG 3350 over a period of 2–3 days at 298 K. The crystals were harvested and cryoprotected in well solution containing 20% glycerol and were flash cooled under liquid nitrogen. Diffraction data were collected on beamline 8.3.1 of the APS (Advanced Photon Source, Argonne National Lab, IL). Data were collected over a rotation range of 180° with an oscillation of 0.5° and 1–2 s exposure per frame, at a crystal-to-detector distance of 300 mm. The data were integrated and scaled using the program HKL2000 [46].

The structure of the GE356 Fab was determined by molecular replacement with the structure of the GE136 Fab (PDB ID: 4KTD) as the search model using the program Phaser in the Phenix suite [47], [48]. The GE136 model was split into two domains, the variable and constant domains for molecular replacement to account for the changes in elbow angle of the Fab. The asymmetric unit comprised two copies of the Fab molecule. Structure refinement was done using Refmac and Phenix, and the model building and adjustment was done using the program Coot [47], [49], [50]. Data collection and final refinement statistics are shown in Table S3.

### Modeling of NHP antibody structure in complex with gp120 core

The ClusPro server 2.0 [51]–[54] was used in the antibody mode [55] to produce structural models of the NHP antibody GE356 in complex with the previously determined crystal structure of the gp120 core in complex with the human antibodies b13 (PDB ID: 3IDX) and F105 (PDB ID: 3HI1). We used the published structures of the non-broadly neutralizing antibodies b13 and F105 based on the criteria that the NHP GE356 antibody displayed a similar binding and neutralizing profile as the human b13 and F105 antibodies. To input the gp120 core structure in the ClusPro server, a separate PDB file was created for the gp120 core from the published structure, removing the human antibody to expose the CD4bs on the core molecular surface. The high-resolution model of the complex resulting from pairing the GE356 antibody and the gp120 core structures was obtained based primarily upon the available alanine scan data that we had determined experimentally.

### Human MAbs

The MAb 2G12 was purchased from Polymun Scientific Inc. The anti-CD4bs MAb F105 was kindly provided by M. Posner (Dana-Farber Cancer Institute), the CD4bs MAbs b6 and b12 were provided by D. Burton (The Scripps Research Institute). HIV-Ig was obtained from the National Institutes of Health AIDS Research and Reference Reagent Program. Soluble CD4 (sCD4 with CD4 D1 to D4 domains) was purchased from Progenics. The CD4-Ig plasmid expression construct was provided by J. Sodroski (Dana-Farber Cancer Institute). VRC01 was previously described [12].

## Results

### Identification of rhesus macaque VH gene segments with high homology to human VH1-2*02

To identify the macaque germline sequence that most closely resembled the human VH1-2 gene segment, we built on our previous study describing the rhesus macaque immunoglobulin loci in comparison to the human immunoglobulin loci [30]. In that study, we demonstrate that the rhesus macaque and human VH gene segments cluster according to the distribution of VH families rather than according to species illustrating their overall homologies. Here, we analyzed the phylogenetic relationship of all known rhesus macaque VH1 open reading frames (ORFs) in the current database [30], in comparison with the human germline gene segment, VH1-2*02, using MUSCLE and PhyML. In addition to the ORFs in this database, we included two rhesus macaque VH1 gene segments that were recently described [56] and are available in Genbank (ABD98406.1 and AER46679.1). Collectively, this analysis identified the rhesus VH1.23 gene segment to have the highest homology to the human VH1-2*02 gene segment, possessing 92.3% homology at the nucleotide level (Fig. 1A). The nucleotide sequence homology between human VH1-2*02 and the other rhesus macaque VH gene segments was between 88.5% and 85.8%. At the amino acid level, the homology between VH1.23 and VH1-2*02 was 89.8% while the other NHP VH1 sequences displayed lower levels of homology (between 87.8% and 77.6%) to VH1-2*02 (Fig. 1B). Of the three amino acids in the heavy chain variable gene segment that define an important motif for VRC01-like antibodies (W50, N58, flanking the HCDR2 region, and R71, highlighted in the red boxes in Fig. 1B), the macaque VH1.23 germline sequences encoded two (W50 and R71). None of the macaque VH1 gene segments identified here contained all three amino acids in the W50/N58/R71 motif.

We next examined the VH usage in the class-switched IgG antibody repertoire of rhesus macaque F128. Memory B cells, defined as CD20^+^, IgG^+^, CD27^+^, CD3^−^, CD8^−^, CD14^−^, IgM^−^ cells, were sorted in 96 well plates at single cell density. After reverse transcription (RT) of the mRNA from the lysed single B cells to generate a cDNA library, a total of 179 sequences were amplified with previously described primers [38], [39] to assess heavy chain VDJ family usage. With the exception of VH6, all other VH family members were detected among these sequences. Focusing specifically on the VH1 family usage, we found that 8.94% of the sequences belonged to the VH1 family and 1.68% of these used VH1.23, demonstrating that this gene segment was expressed in the macaque IgG-switched memory B cell compartment (Fig. 1C).

### VH1.23-using antibodies directed against the HIV-1 CD4bs are elicited by Env immunization

**Figure 2:**
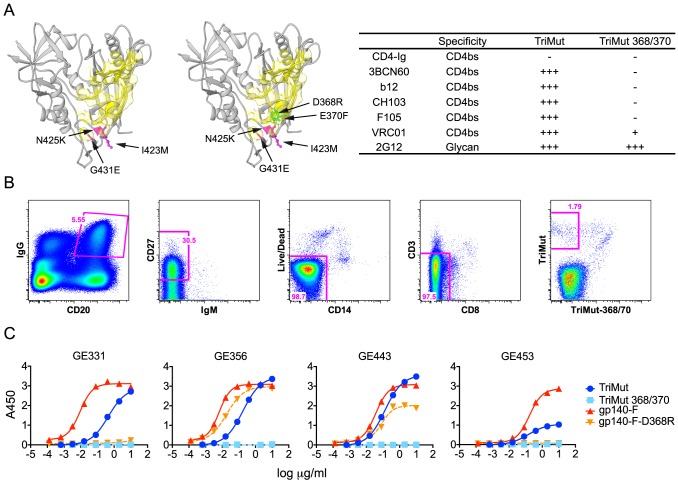
Isolation of HIV-1 gp120 CD4bs-specific vaccine-induced NHP memory B cells, NHP MAbs and their binding specificities. (A) Structural representation of the TriMut and TriMut368/370 probes that were used for sorting CD4bs-specific memory B cells. The key mutations for TriMut (I423M, N425K and G431E) are highlighted in hot pink. The additional TriMut368/370 mutations (D368R and E370F) used for negative selection are highlighted in green and the CD4 footprint is in yellow. The inserted table shows the binding profile of the probes with a panel of CD4bs antibodies and CD4-Ig. Relative binding to the probes was based on the OD450 values at the highest concentration of protein (10 µg/ml) and EC_50_ values: OD450≥3.0 and EC_50_≤0.10 = ++++; OD450≥3.0 and EC_50_>0.10 = +++; 2.0≤OD450<3.0 = ++; 0.2≤OD450<2.0 = +; OD450<0.2 = −. (B) Details describing the flow cytometric sorting of CD4bs-directed memory B cells using the TriMut and TriMut368/370 probes. Gating strategy for sorting IgG^+^ and CD4bs-specific memory B cells; CD20^+^, IgG^+^, CD27^+^, CD3^−^, CD8^−^, CD14^−^, IgM^−^, TriMut and TriMut-368/370 were sorted at single cell density. (C) Four VH1.23-using MAbs (GE331, GE356, GE443 and GE453) were characterized for their capacity to bind trimeric gp140-F+/−D368R and TriMut+/−368/370 by ELISA. Titration curves are shown as Log_10_ dilutions (µg/ml). All antibodies were isolated from the post 2 immunization time point.

From our previous study [32], we established that rhesus macaque F128 was one of the NHPs that displayed the most potent neutralizing titers against a panel of selected viruses (Table S1). To differentially isolate CD4bs-directed memory B cells from the PBMCs of this macaque, we used the TriMut and TriMut386/370 probes. The TriMut pair are based on the gp120 core protein, V3S [36] with three additional mutations in the gp120 bridging sheet region, I423M, N425K, and G431E (Fig. 2A), which eliminate binding to the primary receptor, CD4, without affecting recognition by any of the known CD4bs-directed Abs [57]. The TriMut368/370 probe contains two additional mutations in the CD4 binding loop (D368R and E370F), which specifically eliminate recognition by most CD4bs-directed Abs, including 3BNC60, CH103, and F105, as well as greatly reducing binding of VRC01 by 2 logs of concentration (Fig. 2A and Fig. S1). One advantage of using this gp120 core-based probe pair is that the immunogenic major variable regions 1, 2 and 3 present on gp120 or gp140 Env trimers are genetically deleted from these probes, potentially increasing the efficiency of isolating B cells specific for the CD4bs. In addition, these probes are not recognized by CD4 binding-induced, co-receptor binding site-directed Abs, which are readily elicited by Env immunization of non-human primates [35], making them useful for selective isolation of CD4bs-directed B cells.

Using these probes, CD4bs-directed memory B cells were isolated by sorting CD20^+^, IgG^+^, CD27^+^, CD3^−^, CD8^−^, CD14^−^, IgM^−^, TriMut^+^, TriMut368/370^−^ at single-cell density into 96-well plates (Fig. 2B). The numbers and percentages of the stained and sorted cells are summarized in Table S2. When comparing the cell populations resulting from staining with the TriMut/TriMut368/370 probes, which lack the major variable regions, to those using the gp140-F/gp140-F-D368R trimer probes, that contain the major variable regions used in our previous study [30], we detected a considerably higher percentage of putative CD4bs-specific B cells compared to the total Env pool (50.3% versus 12.6%). Presumably this difference is due to the lack of the V regions and indicates that roughly 50% of B cells directed against the gp120 core recognize the CD4bs. After lysis of the CD4bs-specific B cells, V(D)J transcripts were amplified by RT-PCR [38], [39] and sequenced. To assess the frequency of VH1 usage, we screened a total of 154 heavy chain memory B cell sequences from the TriMut^+^/TriMut367/370^−^ gate and found that 17, approximately 11% of all sequences, used gene segments belonging to the VH1 family. Twelve of these used the VH1.23 gene segment and from this subset we isolated 7 matching heavy and light chains sequences. From these 7 matched pairs, five MAbs were confirmed to be functional after expression: namely GE331, GE356, GE443, GE453 and a clonal relative of GE356, GE460 (not shown).

### Binding specificity and neutralizing activity of the isolated VH1.23-using CD4bs-specific MAbs

To confirm the CD4bs-specific binding characteristics of the VH1.23-using MAbs, we performed ELISA experiments using a panel of Env glycoproteins, including the TriMut, TriMut368/370 cores and the gp140-F, gp140-F-D368R trimers. As expected, all VH1.23-using MAbs bound TriMut probe, but not TriMut368/370, validating the specificity of the flow cytometry-based sorting approach. All four vaccine-induced MAbs also bound the gp140-F trimers while GE356 and GE443, but not GE331 and GE453, recognized the gp140-F-D368R trimeric target glycoprotein. These data indicated that GE331 and GE453 are affected by the D368R mutation in an Env context-dependent manner, or are more dependent upon contacts at the gp120 CD4bs residue 370E for high affinity binding (Fig. 2C). As expected, the human CD4bs-directed human MAbs also demonstrated high affinity interactions with most of the Env variants (Fig. S1A).

The binding characteristics of the VH1.23-elicited MAbs were further analyzed for recognition of selected gp120 Env variants in solution by Bio-Layer Interferometry (BLI), BLI can determine kinetic parameters of Ab association (on-rate, or K_A_) and dissociation (off-rate, *K*_D_) with its epitope as well as binding affinity (KD). All VH1.23 using MAbs displayed high binding affinities for monomeric gp120 as well as to gp120 core and the TriMut, with the exception of GE453 (Fig. S1B and Table S3). Exceptionally rapid on-rates were detected for the interactions of GE331, GE356 and GE443 with both gp120 core and TriMut as shown by the representative binding curves for GE356 (Fig. S1B).

**Figure 3:**
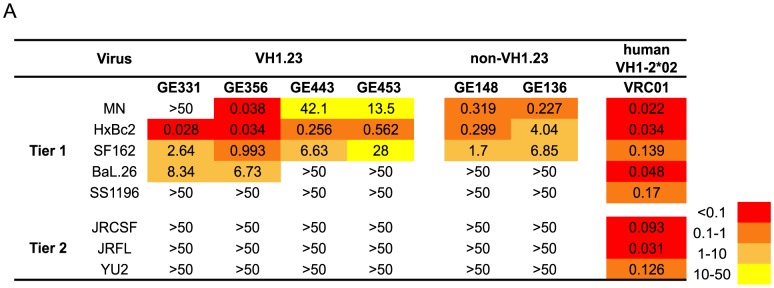
Neutralizing properties of vaccine-elicited CD4bs-directed MAbs. (A) Neutralization activity of four isolated VH1.23-using MAbs from the current study, two previously described non-VH1.23-using NHP MAbs against the CD4bs, and VRC01 were tested against a panel of viruses using the TZM-bl pseudovirus assay. The results are shown as neutralization IC_50_ values, that is, the MAb concentration inhibiting 50% of viral entry. The IC_50_ values are color-coded where red indicates (<0.1 µg/ml), dark orange (0.1–1 µg/ml), light orange (1–10 µg/ml), yellow (10–50 µg/ml) and white (>50 µg/ml).

Next, we analyzed the neutralizing activity of the vaccine-induced VH1.23-using Abs in a standardized neutralization assay [43] against a panel of primary viruses. All MAbs displayed neutralization activity against several tier 1A viruses, including HXBc2, a virus known to be sensitive to CD4bs-directed Abs [58]. Two MAbs, GE331 and GE356, neutralized Bal.26, a virus not neutralized by previously isolated vaccine-elicited CD4bs-directed Abs that utilized VH3 or VH4 gene segments. The potency of GE356 was approximately one log higher than that observed for the other NHP CD4bs-directed MAbs, displaying IC_50_ values of 0.038 against MN, 0.034 against HXBc2, 0.993 against SF162 and 6.73 against BaL.26 (Fig. 3). However, neither of the VH1.23-using MAbs isolated here neutralized the more resistant isolates examined.

### Genetic analysis of the isolated VH1.23-using CD4bs-specific MAbs

**Figure 4:**
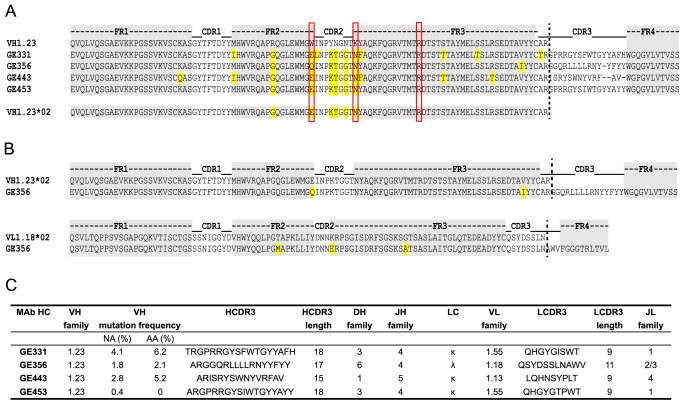
Genetic analysis of the isolated VH1.23-using CD4bs-specific MAbs. (A) Sequence alignments of the isolated NHP VH1.23-using MAbs. The framework regions (FR) are boxed in gray and the amino acid differences compared to the germline VH1.23 sequence are highlighted in yellow. Depicted below the MAb sequences is the newly discovered VH1.23*02 germline allele. (B) Alignments of the GE356 heavy and light chain sequences with the assigned germline sequences. (C) Summary of the genetic characteristics of the VH1.23 vaccine-induced MAbs.

Next, we analyzed the amino acids sequences of the four VH1.23-using MAbs, GE331, GE356, GE443 and GE453, aligned to the reference macaque VH1.23 sequence. Interestingly, we observed that five of the amino acid mismatches compared to the reference VH1.23 sequence were the same in all four MAbs (Fig. 4A), of which at least GE356, GE331 and GE443 are likely to be of independent lineages based on their heavy and light chain V(D)J usages and their HCDR3 identities. We therefore examined the germline VH1.23 sequence in macaque F128, the animal from which the MAbs were cloned, by targeted genomic sequencing and subcloning We selected the reverse VH primers in positions so that only non-rearranged VH1.23 sequences would be amplified from the genomic DNA extracted from the PBMCs and not from V genes that had undergone VH to DJ rearrangement as in a memory B cell where such regions would be deleted (Fig. S2A). By sequencing several independent colonies we detected the existence of two alleles: the previously known VH1.23 allele, which we hereafter refer as VH1.23*01, and a new and previously unidentified VH1.23 allele, hereafter referred to as VH1.23*02 (Fig. S2B). Upon alignment of the MAb sequences to the VH1.23*01 and VH1.23*02 alleles, we conclude that GE356, GE331, GE443 and GE453 originated from the VH1.23*02 allele, yielding an SHM level of the GE356 HC of 2.1% at the amino acid level. Since GE356 was chosen to be studied in detail, we also determined the genomic sequence of its LC, VL1.18. Also here we identified a new allele, VL1.18*02 (Fig. 3C), highlighting the importance of genomic DNA sequencing for correct germline gene assignment and calculations of SHM. Based on these results, we designed a construct encoding the putative germline form of GE356 MAb (Fig. S3A), which upon expression we found bound the gp140-F immunogen illustrating that this MAb had detectable affinity to its ligand also in the absence of SHM (Fig. S3B).

The genetic characteristics of all four VH1.23*02-using MAbs are summarized in Fig. 4C. The average HCDR3 length of the vaccine-elicited MAbs was 17±2 (range 15 to 18), which is similar to the HCDR3 lengths of the infection-induced human CD4bs-directed MAbs [27]. However, the level of SHM calculated for the vaccine-elicited MAbs was considerably lower than the extreme levels of SHM reported for infection-induced CD4bs-directed bNAbs.

### Structural determination and paratope mapping reveal the importance of the GE356 heavy chain CDRs for its interaction with Env

**Figure 5:**
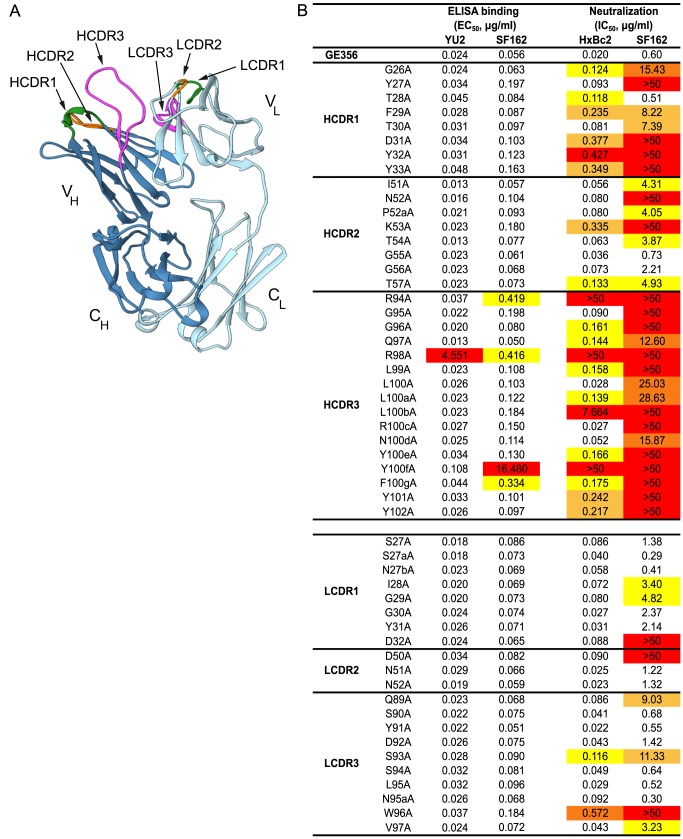
Representation of the GE356 Fab crystal structure and paratope mapping analysis. (A) The GE356 heavy chain is shown in dark blue and light chain in light blue. The HCDR and LCDR loops are colored as follows: CDR1s (green), CDR2s (orange), CDR3s (magenta). (B) Effects of alanine substitutions in the CDRs of the heavy and light chain of GE356 on YU2 and SF162 gp120 binding (left) by ELISA and neutralization capacity (HXBc2 and SF162, right), as measured using the TZM-bl pseudovirus assay, compared to WT GE356. Residues are numbered based on the Kabat numbering system. EC_50_ (Ab concentration for half-maximal binding) and IC_50_ (Ab concentration for 50% neutralization) values are shown. The color-coding reflects fold-decrease in binding or of neutralization activity as compared to WT, in red (>50× WT), dark orange (20–50× WT), light orange (10–20× WT) and yellow (5–10× WT). HCDR3 A93 and LCDR3 A95b were not included in the analysis as there were alanines in these positions in the WT sequence.

To obtain a more detailed understanding of the properties of the vaccine-induced, VH1.23-using MAb GE356, we crystallized the Fab fragment of this MAb using high-throughput crystallization screening and solved its structure (crystallographic details, Table S4). The Fab displayed the typical antibody characteristics of proximal light and heavy chains, CDR loops clustered at the putative binding site and beta-sheet interactions in the constant domains (Fig. 5A). The availability of this high resolution crystal structure facilitated identification of critical contact residues of the most potent VH1.23 using MAb, that is, the paratope interactions of GE356 with HIV-1 Env using several convergent approaches. GE356 functional capacities were analyzed by an extensive alanine scan of its heavy and light chain CDRs. A panel of GE356 alanine mutants was generated and tested for binding or loss thereof to monomeric YU2 or SF162 gp120 by ELISA in comparison to wild-type (WT) GE356 interaction. The ELISA results showed very little impact of single alanine substitutions in the GE356 CDRs on recognition of YU2 gp120 with the exception of an arginine to alanine substitution at position 98, in which binding was significantly reduced. Three additional positions (R94, Y100f, and F100g) also decreased binding to SF162 gp120 upon substitution with alanine, indicating involvement of these residues in GE356 to Env gp120 interaction (Fig. 5B, left).

A markedly different result was obtained when we assessed the impact of the alanine substitutions on the ability of GE356 variants to neutralize selected HIV-1 strains. We assessed neutralization activity of the GE356 alanine mutants against the HXBc2 and SF162 viruses relative to the potency of WT GE356 MAb. HXBc2 was potently neutralized by GE356 with an IC_50_ of 0.02 µg/ml, whereas SF162 was neutralized with a lower IC_50_ potency of 0.60 µg/ml (Fig. 5B, far right). In general, compared to the ELISA, more alanine substitutions in the GE356 CDRs impacted neutralization of HXBc2. Substitutions in all three CDRs of the heavy chain resulted in a negative impact on neutralization whereas alanine substitutions in the light chain CDRs had little or no impact on the neutralizing capacity. We performed a similar scan against the SF162 virus and found that every alanine substitution in the heavy chain CDR3 region and all, with the exception of residue 28, in the heavy chain of CDR1 region negatively affected GE356 neutralizing capacity. The three residues that resulted in complete elimination of HXBc2 and SF162 neutralization (R94A, R98A, and Y100fA) corresponded to three of the four residues that substantially decreased binding to SF162 gp120. The alanine scan of the light chain CDRs showed that a few additional alanine substitutions negatively affected neutralization of SF162 when compared to HXB2, consistent with the lower neutralization potency of WT GE356 for the SF162 virus.

To further define the interaction between GE356 and Env, we mapped the epitope for GE356 within the CD4 binding region of gp120. We used a panel of JRCSF gp120 Env mutants containing single alanine point mutations in gp120 to perform this focused scan (Table S5) [44]. Residues were selected based upon the criteria that they were known contact residues of CD4 or the CD4bs-directed Abs with gp120 [26], [45], [59]–[61]. For GE356, the mutational analyses showed that one substitution in the CD4 binding loop, one in the β19 sheet and one in the α5 region considerably diminished GE356-mediated recognition of gp120. When compared to VRC01 epitope mapping performed in parallel, we saw that different residues affected recognition, suggesting a qualitative difference in the binding specifics between these antibodies within the CD4bs.

**Figure 6:**
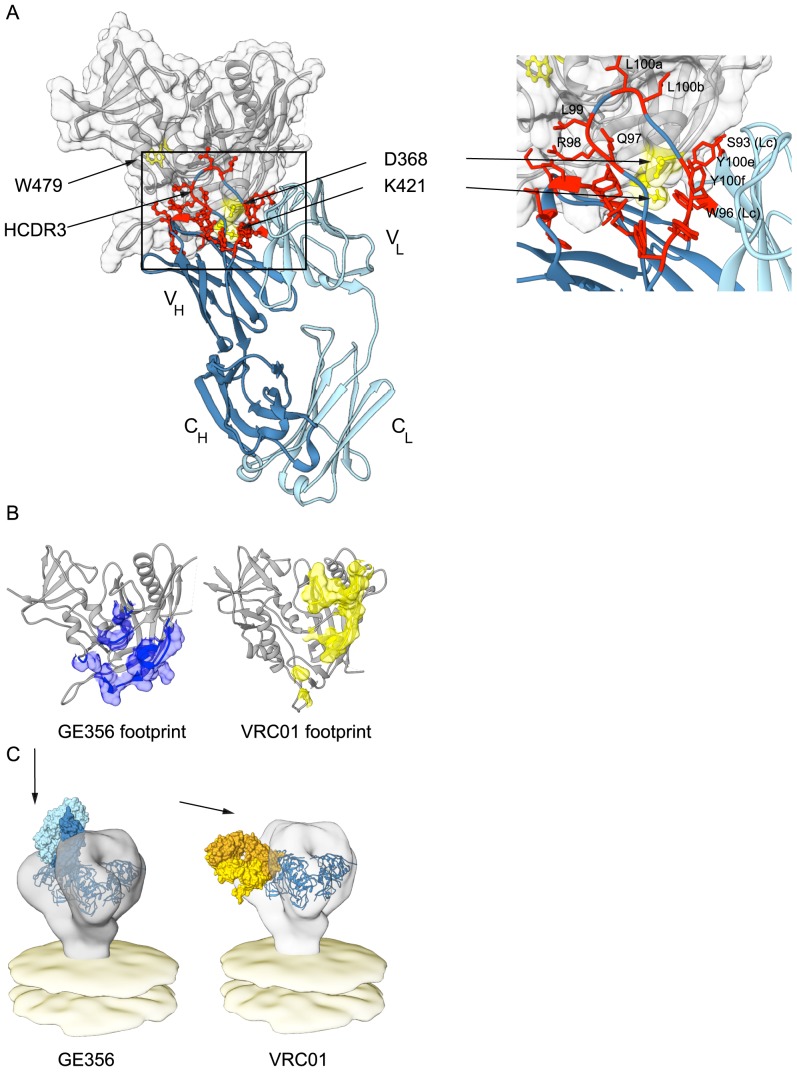
Mapping GE356 binding to gp120, modeling of the GE356:core gp120 complex and fitting to the trimer density. (A) A panel of JRCF Env mutants containing single Ala point mutations in gp120 was used for a limited alanine scan (Table S5). The three most critical residues revealed by this scan are highlighted on the structure of core gp120 (gray) in yellow and labeled as indicated. A ClusPro model of GE356 in complex with core gp120 was derived from these data and is shown with heavy (dark blue) and light (light blue) chains, docking to the gp120 core. The most critical residues that affect HXBc2 and SF162 neutralization in the antibody paratope from the alanine scan are shown in panel Fig. 5B are mapped onto the structure of the GE356 MAb and are indicated in red. The boxed area shows an expanded view of the most crucial modeled interactions. Residues from the light chain are indicated (LC). (B) Footprint of GE356 (left) compared to VRC01 (right) on the gp120 core structure (gray cartoon representation). The GE356 footprint (blue surface) was obtained by modeling GE356 binding to the b13-bound gp120 crystal structure (PDB ID: 3IDX) with ClusPro 2.0. The VRC01 footprint (yellow surface) on the gp120 core is derived from the crystal structure of the complex (PDB ID: 3NGB). (C) Left, a model of the mode of recognition of GE356 (light chain in light blue and heavy chain in dark blue) by fitting of the GE356:gp120 core into the cryo tomographic density of the unliganded HIV-1 spike [67]. Right, similar fitting of the VRC01 (light chain in light yellow and heavy chain in gold):gp120 core into the viral trimer density (off white).

Based upon the combined analyses of the MAb paratope, the epitope on gp120 and the crystal structure of the GE356 Fab described above, a model of interaction between GE356 and gp120 was generated using ClusPro 2.0 (Fig. 6A). This model was consistent with indications that most of the residues implicated in GE356:gp120 interaction were located at or near the protein∶protein interface. Two residues implicated by the alanine scan are located at the base of the HCDR3 (R94 and R98), slightly distal to the modeled protein interface, and likely contribute to HCDR3 stability by side chain interactions at the base of the extended loop. When we compared GE356 to two VH4-using macaque MAbs, GE136 and GE148, we found that all three MAbs rely heavily on their HCDR3s for binding. All have a hydrophobic tip that extends towards gp120 and all three MAbs have a basic residue (R98 in GE356 and K100e in GE136/GE148) near the tip of the HCDR3 that may form a hydrogen bond with the acidic D368 in the CD4 loop of gp120. GE136/GE148 display the usual two beta strands that often form the base and sides of the HCDR3 to provide loop rigidity, while GE356 has a beta stand only on one side, which may result in greater flexibility of this HCDR3 (Fig. S4).

Using the coordinates derived from the ClusPro model-building, the footprint of GE356 on the gp120 core was approximated using Chimera (Fig. 6B). The footprint suggested that GE356, like the VH3- and VH4-using, vaccine-elicited macaque MAbs [30], mapped further towards the gp120 core bridging sheet compared to the crystal structure of VRC01 in complex with the core. This relatively subtle difference in the footprints of the two MAbs translates into a marked difference of binding orientation when the MAb:core complexes were fit into the cryotomographic density of the trimeric HIV-1 spike, a process that we have previously validated [33] (Fig. 6C). By this analysis we observed that GE356 attempts to access the CD4bs by a more vertical angle of access than does VRC01, an angle that is likely not permitted on the functional spike. We also performed the same procedure using the recently reported high resolution structure of the BG505 SOSIP trimer [62] with a similar outcome (Fig. S5). The difference in angle of approach between the bNAb VRC01 and the non-bNAb GE356 likely explains the large difference in their capacity to neutralize, or not, more resistant HIV-1 isolates. The limited neutralization capacity of GE356 is likely due to clashes with the closed variable region cap that exists on the top of the primary isolate spike as it attempts to access the CD4bs from a vertical angle of approach relative to the viral membrane in a manner similar to that recently described for the VH4-using macaque CD4bs-directed MAbs, GE136 and GE148 [33].

## Discussion

The now well-established paradigm that some chronically HIV-1 infected individuals develop bNAbs has direct translational value as the epitopes of such Abs may be used as templates for Env-based immunogen design [63]. A striking finding from genetic and structural studies of one class of bNAb represented by the CD4bs-directed MAbs, VRC01 and 3BNC60, is that despite independently evolving in different HIV-1 infected individuals, Abs of this class use the same germline VH1 allele, VH1-2*02, and display remarkably similar structural features and modes of interaction with HIV-1 gp120 [9], [12], [13], [26]–[28]. However, VH1-2*02 gene segment usage alone is not sufficient to generate Abs with bNAb properties, as these Abs also use light chains with unusually short CDR1 and CDR3 regions, features that are critical for their efficient interaction with the native spike to mediate broad neutralization [26], [27], [29]. In addition, these Abs acquire a high degree of SHM in both the CDRs and frame work regions (FWRs), which contributes to their remarkable breadth and potency [64]. Nevertheless, the selective use of the VH1-2*02 gene segment by multiple VRC01-like Abs suggests that HIV-1 Env vaccine candidates should be evaluated in hosts encoding this or highly homologous germline sequences, such as in transgenic animals encoding the human Ig locus or in NHPs shown to have an Ig locus that is highly homologous to humans.

We demonstrated previously that rhesus macaques, which have an overall sequence homology to humans of approximately 93% [42], encode germline Ig heavy and light chain variable gene segments that cluster together in families with the corresponding human gene segments [30]. Here, we identified the closest reported rhesus macaque homologue to VH1-2*02 to be VH1.23. Despite the relatively infrequent presence of VH1.23 in the recombined V(D)J repertoire of IgG-switched NHP memory cells, we were able to isolate a panel of clonally distinct CD4bs-directed VH1.23-using MAbs. One of these MAbs, GE356, displayed increased neutralizing potency compared to previously isolated vaccine-elicited CD4bs-directed MAbs [30] without an improvement in neutralization breadth of primary HIV-1 isolates. We demonstrated by genomic sequencing that this MAb arose from the activation of a newly defined rhesus macaque VH1.23 allele termed VH1.23*02.

Several features of GE356 distinguishes it from VRC01-like Abs, including the use of a light chain with a CDR3 length of 11 amino acids and a SHM level of 3.1%, which is considerably less than VH1-2*02-using bNAbs isolated from chronically HIV-1 infected individuals. Thus, despite using highly homologous VH gene segment orthologues, GE356 and VRC01 bear little resemblance. The difference in SHM between vaccine-elicited and infection-induced Abs is not surprising given that the average time for bNAbs to develop in infected individuals is around 2.5 years. These Abs evolve in response to persistent and ever-changing antigenic challenge and chronic immune activation, in contrast to the transient stimulation with an invariable antigen in adjuvant from the prime-boost vaccination regimen used here.

To define structural features of the VH1.23-using GE356 MAb, we performed an extensive Ala-scan of the CDR regions of the HC and LC and crystallized the Fab to determine its structure to permit additional functional mapping and modeling analysis. The Ab paratope scan revealed that all three HCDRs, but in particular HCDR3, contributed to Ab function in contrast to VRC01, which interacts with gp120 independently of its HCDR3 except through one contact residue from this region [61]. We conclude that GE356 behaves like most typical MAbs, for which the HCDR3 predominantly provides most of the binding energy. By ClusPro-mediated docking of the GE356 Fab structure onto the gp120 core, the interactions between the HCDRs and the cognate epitope on gp120 were confirmed. The model was consistent with the involvement of residues revealed as contributing to binding energy or HCDR3 stabilization as defined by the alanine scanning. The LCDRs were less involved with binding at the protein∶protein interface, consistent with the results from the paratope scanning.

The isolation of GE356 and other CD4bs-directed MAbs described herein demonstrate that Env trimer immunization activates B cells encoding a macaque orthologue of the human VH1-2 gene segment. Future studies using Env immunogens that better mimic the native HIV-1 glycoprotein spike will be important to attempt to elicit Abs with a greater breadth of neutralization. Furthermore, the structure-based design of Env immunogens that bind the putative VRC01 germline sequence represent an interesting alternative strategy to elicit this highly effective class of antibodies [56], [65]. However, despite rapid advances in immunogen design, the elicitation of VRC01-like antibodies is likely to be a major challenge due to the special features of these antibodies. The studies presented here suggest that a more achievable goal for the HIV-1 vaccine field may be to elicit CD4bs-directed Abs such as CH103, a bNAb that relies on the HCDR3 loop for interacting with its epitope [66] and approaches the functional spike from a more vertical angle of access than does VRC01 [33]. CH103 displays about 13% SHM in its VH region and possesses no insertions or deletions and is therefore overall more similar to the vaccine-induced Abs, including the GE356 MAb described here. Additional MAbs that use their HCDR3s for binding to HIV-1 Env and possess broadly neutralizing activity isolated from chronically infected individuals are forthcoming (John Mascola, personal communication). Such studies are encouraging, given the important role of the HCDR3 in antigen recognition by antibodies in general. Future studies defining the fine specificities of CD4bs-directed Abs elicited by next generation Env immunogens will be central to our understanding of vaccine-induced responses aimed to elicit neutralizing Ab responses against HIV-1.

## Supporting Information

Figure S1 **Binding specificities of human MAbs.** (A) The human MAbs were characterized for their capacity to bind trimeric gp140-F+/−D368R and TriMut+/−368/370 by ELISA. Titration curves are shown as Log_10_ dilutions (µg/ml). (B) The right panel shows binding affinities of the VH1.23-using NHP MAbs to gp120, gp120 core and TriMut were analyzed by BLI using an Octet RED96 system with the MAbs immobilized on the chip surface and the Env ligands as the analyte. Several selected human infection-induced MAbs were also examined for comparison and the dissociation constants (*K_D_*) between these different MAbs and the ligands are shown. The left panels show the kinetics of binding of GE356 to gp120, gp120 core and TriMut at different concentrations of the each ligand.
(EPS)

Figure S2 **Primer design and alignments of germline VH protein sequences from genomic DNA sequencing.** (A) The primer design for determination of VH1.23 sequences are shown with leader sequences (blue) and the coding VH sequence (red). To ensure amplification of VH1.23 sequences arising from non-rearranged germline DNA, and not recombined V(D)J sequences, we performed PCR using two independent primer pairs in which the reverse primers aligned to sequences in the genome that would be deleted upon VH to DJ rearrangement; PCR1: MMUL VH1.23 fw primer (forward) was used in combination with MMUL VH1.23 rev primer (reverse) and PCR2: 5′VH1A.se (forward) was used in combination with VH1.23 AS-A (reverse). The PCR fragments from each of these reactions were inserted into cloning vectors and several individual colonies were picked for sequencing. (B) Amino acid sequence alignments of germline VH gene segments from several isolated clones resulting from genomic sequencing. PCR1A and PCR1B refers to independent PCRs performed with the same primers, PCR2 refers to reactions performed with a second set of primers (see A). Amino acid differences compared to the germline VH1.23 sequences are highlighted in yellow. The remaining amino acid differences in GE356 compared to VH1.23*02 are highlighted in green.
(EPS)

Figure S3 **Design of putative germline GE356 and binding specificity.** (A) Mature and putative germline GE356 sequences; heavy and light chain V regions (gray), heavy chain D region (white) and heavy and light J regions (green). CDR3 sequences are typed in red. (B) The binding specificity of the putative germline version of GE356 and the mature GE356 to gp140-F trimers immobilized on the ELISA plate. Titration curves are shown as Log_10_ dilutions (µg/ml).
(EPS)

Figure S4 **Comparison of macaque MAbs GE356, GE136 and GE148 binding to core gp120.** (A) Model of GE356 binding to core gp120 with GE136 and GE148 superimposed onto GE356. GE356 (blue), GE136 (rose), GE148 (green), core gp120 (gray). The core is in the GE356-docked model conformation. (B) Magnified view of the GE356, GE136 and GE148 HCDR3s. (C) Magnified view of the GE356 and GE148 HCDR3s with key residues shown to play a role in Env binding or neutralization by the Ala scan analysis highlighted in red (stick representation).
(EPS)

Figure S5 **Superimposition of the GE356:gp120 core model into the crystal structure of the soluble BG505 SOSIP.664 trimer.** A theoretical docking of the GE356 Fab:gp120 core into the SOSIP trimer. The overlapping translucent area between GE356 and SOSIP reveals an area of clash between the two proteins (to the left of the gray arrow), and is consistent with the inability of GE356 to neutralize tier 2 resistant isolates due to the attempted vertical approach to the CD4bs epitope. The GE356 light chain is shown in light blue and the heavy chain in dark blue. The BG505 SOSIP.664 trimer (off white) from PDB ID:4NCO is shown from which the trimer-bound PGT122 Fabs from the published crystal structure [62] were removed for clarity.
(EPS)

Table S1 **Neutralizing activity in the plasma of animal F128.** Plasma from rhesus macaque F128, collected post-2 and post-5 inoculations with YU2 gp140-F trimers, were tested for neutralizing activity against a cross-clade pseudovirus panel using the TZM-bl assay. Neutralization ID_50_ values (i.e. plasma dilution at which 50% of viral entry is inhibited) are shown.
(EPS)

Table S2 **Properties of flow cytometric sorts.** CD4bs-directed memory B cells from the PBMCs of macaque F128 were isolated by sorting CD20^+^, IgG^+^, CD27^+^, CD3^−^, CD8^−^, CD14^−^, IgM^−^, TriMut^+^, TriMut368/370^−^ cells. The numbers and percentages of the stained and sorted cells are summarized.
(EPS)

Table S3 **Binding affinities of vaccine- and infection-elicited MAbs to a panel of Env ligands.** Binding affinities of the VH1.23-using NHP MAbs, along with several human infection-induced MAbs, to gp120, gp120 core, and TriMut were analyzed by BLI using an Octet RED96 system with the MAbs immobilized on the chip and the Env ligands as the analyte in solution. The dissociation constants (*K_D_*), on-rates, and off-rates are shown.
(EPS)

Table S4 **Crystallographic collection and refinement statistics.** The Fab fragment of the VH1.23-using NHP MAb, GE356, was crystallized and its structure solved. Data collection and final refinement statistics are shown.
(EPS)

Table S5 **Mapping GE356 Ab binding to gp120 by alanine-scanning the gp120 CD4bs.** The epitope of GE356 within the gp120 CD4bs was mapped using a panel of JRCSF pseudovirus variants containing single alanine point mutations in gp120 within the CD4bs to assess binding affinities relative to wild type (WT) gp120. The CD4bs-directed bNAb, VRC01, was also tested for comparison. Binding by the bNAb 2G12 was used to normalize gp120 expression levels, with exception of the N332A mutant for which HIV IgG was used instead of the 332N-sensitive 2G12. Relative binding affinities were calculated as described in Materials and Methods. Mutations resulting in a three-fold reduction in binding of the MAbs relative to WT gp120 are highlighted in yellow.
(EPS)
